# Hemifacial spasm: an update on pathophysiology, investigations and management

**DOI:** 10.1007/s00415-025-13220-y

**Published:** 2025-07-10

**Authors:** Aaron Jesuthasan, Ammar Natalwala, Indran Davagnanam, Tabish Saifee, Ludvic Zrinzo

**Affiliations:** 1 Department of Neurology, Addenbrooke’s Hospital, Cambridge, CB2 0QQ UK; 2 Unit of Functional Neurosurgery, National Hospital for Neurology and Neurosurgery, University College London Hospitals NHS Trust, Queen Square, London, WC1N 3BG UK; 3 National Hospital for Neurology and Neurosurgery, University College London Hospitals NHS Trust, Queen Square, London, WC1N 3BG UK; 4 Department of Brain Repair and Rehabilitation, UCL Queen Square Institute of Neurology, Queen Square, London, UK

**Keywords:** Hemifacial spasm, Movement disorders, Neurovascular conflict, Botox, Microvascular decompression

## Abstract

Hemifacial spasm (HFS) is characterized by involuntary, paroxysmal contractions of muscles innervated by the facial nerve that can lead to a negative impact on daily activities, including reading or driving, as well as psychosocial well-being. HFS remains a clinical diagnosis with characteristic features, however investigations can be immensely helpful. MRI, particularly heavily weighted T2 high-resolution sequences, continues to be invaluable to assess for structural abnormalities, including the presence of neurovascular conflict (NVC) in the cerebellopontine angle. The NVC, as well as its mechanistic roles in HFS, has been better characterized in recent studies, as will be summarized in this update. We will highlight the importance of MRI reporting, as false negatives may lead to delays in neurosurgical referral, while over-reporting of incidental findings can lead to inappropriate intervention and/or treatment failure. This point is reinforced by findings from recent studies, which advocate for the use of 3D techniques to further improve MRI reporting and ultimately patient outcomes. Moreover, recent investigations have shown that botulinum toxin and microvascular decompression are highly effective treatment options for HFS and additionally suggest factors that may further influence the outcomes with these interventions. The recent use of pulse radiofrequency ablation and electroacupuncture may provide alternative avenues of treatment, alongside oral medications, that can be used in HFS but require significant refinement to improve their overall efficacy, durability and safety.

## Introduction

### Epidemiology

Hemifacial spasm (HFS) is a movement disorder characterized by involuntary, paroxysmal contractions of muscles involved in facial expression, which are innervated by the ipsilateral facial nerve. A recent study of the Finnish population suggested the mean annual age-standardized incidence of HFS is 1.53 per 100,000 individuals, with a 2:1 predilection for women over men (1.94 vs 1.05 per 100,000, respectively) [1]. The estimated worldwide prevalence is approximately 14.5 per 100,000 for women and 7.4 per 100,000 for men [2]. Disease onset usually occurs from the fourth through to the sixth decades of life [1, 3]. HFS is often sporadic and unilateral, but in 2.6% of cases may be bilateral [3]. Familial cases are seldom reported in the literature, and account for 2–3% of all HFS cases [4].

### Etiology

It is well established that an ectatic or aberrant arterial loop (typically the anterior-inferior cerebellar artery, posterior-inferior cerebellar artery, or vertebral artery) interacts with facial nerve fibers in the posterior fossa to induce HFS [5–17]. However, recent studies have enabled further characterisation of this neurovascular conflict (NVC) to provide greater insight into the pathophysiology and enhance pre-operative planning.

Radiological NVC is now classified as being either absent (discernible CSF between vessel and nerve), contact present (vessel merely touches the nerve but there is no discernible CSF between vessel and nerve), or deformity present (vessel displaces/compresses the nerve) [18]. Furthermore, the facial nerve can be divided into four anatomical segments: the attached segment (AS), root detachment point (RDP), proximal cisternal segment (PCS) and distal cisternal segment (DCS), providing a useful framework to describe the location of NVC along the nerve [18–21].

It is important to note that NVC in asymptomatic patients is common. Furthermore, a recent study by Traylor et al. (2021) involving 330 patients with HFS showed that NVC was found in 97.9% of symptomatic and 38.8% of asymptomatic cases [22]. NVC in the proximal cisternal segment was more common on the symptomatic (96.4%) than the asymptomatic side (12.7%), with deformity also being more commonly noted on the symptomatic (70.3%) than the asymptomatic side (1.8%). This suggests that NVC resulting in deformity, particularly in the proximal portion of the facial nerve is highly associated with HFS. Li et al. (2024) examined 214 patients and reported that NVC of any severity is statistically more common along the AS, RDP, and PCS of symptomatic versus non-symptomatic nerves. However, at the cisternal part of the facial nerve, only NVC associated with deformity, and not contact, was sufficient to cause HFS [18].

Various theories have recently been proposed to explain these findings without being mutually exclusive. The facial nerve is relatively fixed at the AS, RDP and PCS and is therefore susceptible to structural damage by arterial pulsations. Moreover, at this point, nerve fibers at the AS are only covered by an arachnoid membrane, without any epineurium or connective tissue septa between the nerve fascicles [3, 23]. The PCS is also the zone of transition between central and peripheral myelin, further increasing its susceptibility to damage. Structural damage of the facial nerve can subsequently result in local demyelination, leading to increased nerve excitability and ephaptic transmission. Alternatively, rhythmic pulsing on sensitive facial nerve axons may cause chronic antidromic signaling and hyper-excitability of the facial motor nucleus [24, 25].

These mechanisms add to a previous theory, which implicated sympathetic nerve fibers surrounding posterior fossa vessels in the pathophysiology of HFS, and suggested that arterial pulsations may damage the vessel tunica adventitia and nerve epineurium, to allow “crosstalk” between exposed sympathetic nerve fibers and facial nerve fibers [26]. This was supported by data from preclinical rodent models, which demonstrated that electric stimulation of nerve fibers on the offending artery wall led to a facial muscle response, while their subsequent chemical blockade led to temporary cessation [26]. Furthermore, resection of the sympathetic ganglion in these models led to permanent disappearance of the abnormal facial muscle response, suggesting that sympathetic nerves may bridge the cross-transmission between facial nerve fibers.

### Natural history and impact of HFS

In the absence of treatment, there remains significant variability in the reports of patients obtaining spontaneous relief of symptoms. Earlier studies suggested that only a small minority of patients (2.3–4.2%) achieve symptom control without botulinum toxin therapy or microvascular decompression [27–29]. However, a more recent study involving 104 HFS patients with an average follow-up period of 12 years showed that 41.3% of patients were in remission, either with little (13.4%) or no spasm (27.9%), for 2 months to 23 years (6.4 years on average) without treatment [30].

The age of symptom onset is reported in a very recent study to be a significant factor influencing the progression of HFS, with an age of onset > 45 years correlating with more rapid disease progression (worsening severity on the Samsung Medical Center Grading System) [31]. One theory, albeit with little evidence, suggests that this may be due to delayed remyelination with increasing age [31]. This is additionally thought to be the reason why individuals with the APOE ε4 allele display a faster HFS disease course, as shown by a recent study [32]. It has also recently been reported that compression of the proximal cisternal segment of the facial nerve associated with brainstem deviation leads to more rapid disease progression [31]. Conversely, other factors including gender, the presence of hypertension or diabetes mellitus, laterality of symptoms, the number of offending blood vessels and the involvement of the vertebral artery were not found to significantly influence the rate of disease progression [31].

## Investigations

Investigations can be immensely helpful to exclude potential HFS mimics, particularly when the distinguishing clinical signs are absent or very subtle. For example, epileptiform discharges may be revealed on electroencephalography (EEG) to indicate seizure-related activity. Electromyography (EMG) may also show short 5–150 Hz bursts of motor unit potentials, that appear as doublets, triplets or multiplets, to support a diagnosis of myokymia [33].

Neuroimaging, in the form of MRI, remains invaluable to assess for potential structural causes, such as vestibular schwannomas, meningiomas, or arteriovenous anomalies. High-resolution T2-weighted sequences with MRA may also be able to demonstrate an area(s) of NVC [18, 34]. However, this may not always be clear, for example if the thin sections do not capture the vessel or nerve’s origin, or the patient possesses a very small caliber of the contributing vessel, a small volume posterior fossa or crowded cisternal contents [34].

Recent studies have therefore advocated for the use of three-dimensional time-of-flight MRA (3D-TOF MRA) and three-dimensional fast imaging employing steady-state acquisition (3D-FIESTA) at 3 T to better characterize a causative NVC and optimize post-surgical outcomes in both HFS and trigeminal neuralgia [18, 35]. 3D-TOF MRA enhances the contrast imaging of the blood flow and static organization. 3D-FIESTA additionally provides imaging of the outside of the vessels even in the absence of blood flow and displays images of the vertebrobasilar and nerve systems surrounded by CSF. As demonstrated very recently by Li et al. (2024), these modalities also allow the reconstruction of axes to better identify and assess an area of NVC [18]. A study has shown that the combination of 3D-TOF MRA and 3D-FIESTA identified surgically-verified NVCs in 35/36 symptomatic nerves of trigeminal neuralgia patients [36]. For HFS, the accuracy of identifying the offending vessel was recently shown to be 91.1%, 77.8% and 76.9% for the superior, anterior-inferior and posterior-inferior cerebellar arteries respectively [35]. However, in the case of multiple vessels being involved, the accuracy was only 30%. In general, it remains exceedingly rare to not visualize an NVC in the context of HFS.

It should also be emphasized that the interpretation of MRI in HFS patients relies on a radiologist or neurosurgeon being familiar with the anatomical details of the facial nerve root. In our experience, initial MRI reports may often overlook the compression at the attached or cisternal segments of the facial nerve, or inaccurately implicate an incidental neurovascular contact. This is particularly highlighted by a recent study, which investigated refractory HFS patients who failed to respond to microvascular decompression [37]. On repeat MRI, persistent areas of NVC were identified along the facial nerve, proximal to the previous surgical site. This was thought to be partly attributable to suboptimal interpretation of the pre-operative MRI [37].

## Management

It is important to note that although HFS may produce cosmetic and task-disruptive symptoms, it is still not a life-limiting condition and many patients subsequently choose non-operative management, avoiding surgical interventions that carry small but significant risks. However, for patients who find the symptoms sufficiently distressing to impact on quality of life, pharmacological or surgical treatments may be sought [38–40].

### Oral medications

Oral medications remain a commonly trialed method of treatment for HFS patients, particularly for those who are deemed unsuitable or unkeen to try botulinum toxin or surgical intervention, although the clinical benefit is only observed in a small minority of individuals [41]. The medications used typically include anti-spasticity and antiseizure medications; however, the evidence for many of these medications is often based on isolated case reports. Several medications (e.g. carbamazepine, baclofen, pizotifen and clonazepam) also produce unwanted side effects especially in the elderly population, such as somnolence, dizziness, ataxia and weakness. However, gabapentin and levetiracetam may be better tolerated [42–44]. The therapeutic mechanisms underpinning these medications in HFS remain unclear, but may relate to  reducing hyper-excitability of the facial nerve and its nucleus by blocking voltage-sensitive sodium and T-type calcium channels, reducing excitatory glutamatergic transmission or enhancing GABAergic neurotransmission [45].

### Botulinum toxin

Botulinum toxin A, continues to produce a significant improvement in symptoms in the majority of patients (efficacy ranging from 73 to 98.4%) and occasionally leads to spontaneous resolution of symptoms [27, 46]. A recent study has therefore implied that it is used in up to 90% of HFS patients [1].

Adverse effects are sometimes reported, with a recent study suggesting that ptosis and facial weakness are the most commonly observed, although these are generally mild and transient if present [46, 47]. A recent multivariate analysis of factors influencing the short-term prognosis of HFS after botulinum toxin suggested that hypertension was an independent risk factor for poorer prognosis [48]. This could be viewed as concerning, as many patients with HFS often have co-existing hypertension [32, 49]; however, the majority of these patients still respond significantly well to botulinum toxin. The long-term efficacy of botulinum toxin also remains promising, as a previous study suggested that it continues to be effective for up to 10 years after its initial commencement in HFS patients [28]. A recent study has supported this, showing that long-term use was safe and effective for HFS, regardless of underlying etiology [47].

Immunoresistance to botulinum toxin remains rare due to the low dosages often utilized for HFS. However, the ultimate wearing off from the effect still requires patients to attend the clinic on a 3- to 6-monthly basis for repeated botulinum toxin injections, which may not always be a feasible option due to frailty status or personal commitments. The botulinum toxin injections also necessitate appropriately skilled and knowledgeable clinicians to select the optimum dosages and precisely identify the target muscles, posing greater logistical challenges than oral medications.

### Surgical management

Microvascular decompression (MVD) remains the only potential curative treatment for HFS, producing a significant benefit in up to 90% of patients who undergo an operation [50]. However, it has recently been suggested in certain groups that only 10% of HFS patients are ultimately referred for MVD [51], with a mean delay from diagnosis to surgery of up to 5 years [1]. It is, therefore, essential that clinicians are aware of this highly efficacious treatment option for HFS patients and refer them in timely fashion.

MVD usually involves a standard retrosigmoid craniectomy, with or without adjunctive endoscopy, to provide adequate intracranial visualization and achieve atraumatic decompression of the facial nerve from the culprit blood vessel(s) [52–54]. While the details of the procedure are explained extensively elsewhere [51], the culprit vessels are preferably mobilized or transposed away from the cisternal segment and point of maximum compression, rather than solely interposing implant material (e.g. Teflon) between the nerve and vessels. Insertion of implant material remains a much debated area in the context of HFS, as certain studies have suggested that it leads to poorer outcomes due to improper placement or using an inappropriate size of material [55], and can lead to the formation of adhesions to the facial nerve, thus perpetuating HFS or leading to a delayed facial weakness. [56–59]. However, it is still common practice in many centers.

It is increasingly recognized that there may be a time delay before the effects of MVD are fully noticed (in up to 30% of cases), amounting to approximately 30 days in the majority of patients but can be up to a year in a minority [50, 60, 61]. A recent analysis of factors related to delayed cure of HFS following MVD implied that non-transposition was significantly associated with a delayed effect [61]. In contrast, age, gender, laterality, number of offending arteries, vertebral artery compression, number of compression sites, compression at the cisternal segment, pre-operative botulinum toxin treatment and indentation of the brainstem on pre-operative MRI were not significantly related to delayed effects [61].

A very recent study suggested that the use of botulinum toxin as a treatment pre-operatively does not significantly alter surgical outcomes, and patients who are often refractory to botulinum toxin injections achieve a similar curative rate [62]. This adds to previous findings, which proposed that gender, laterality of symptoms, disease duration and severity are also unrelated to treatment response [63, 64]. However, there may be a higher risk of a delayed facial palsy post-operatively in patients with more severe HFS when compared to patients with milder disease at baseline [63].

The long-term results of MVD remain mixed, with a previous study (that assessed results from > 780 MVD procedures) suggesting that up to 9% of patients may require a re-operation [65]. However, a recent meta-analysis demonstrated that spasm freedom following a re-operation is shown to be less successful at achieving symptom relief than a first operation [50]. Re-operations were also shown to be associated with a greater number of complications than a first procedure, with nervous system lesions shown to potentially rise in likelihood from 10.6% with the initial surgery to 20.7% following a revision. The optimum timing of a re-operation in relation to the initial procedure is not fully clear, as it has been suggested that it would be reasonable to wait for up to a year to assess if the initial procedure has provided a significant relief of symptoms [60]. However, other sources have suggested that extending the interval between operations to more than 30 days is more likely to result in treatment failure [50].

**Fig. 1:**
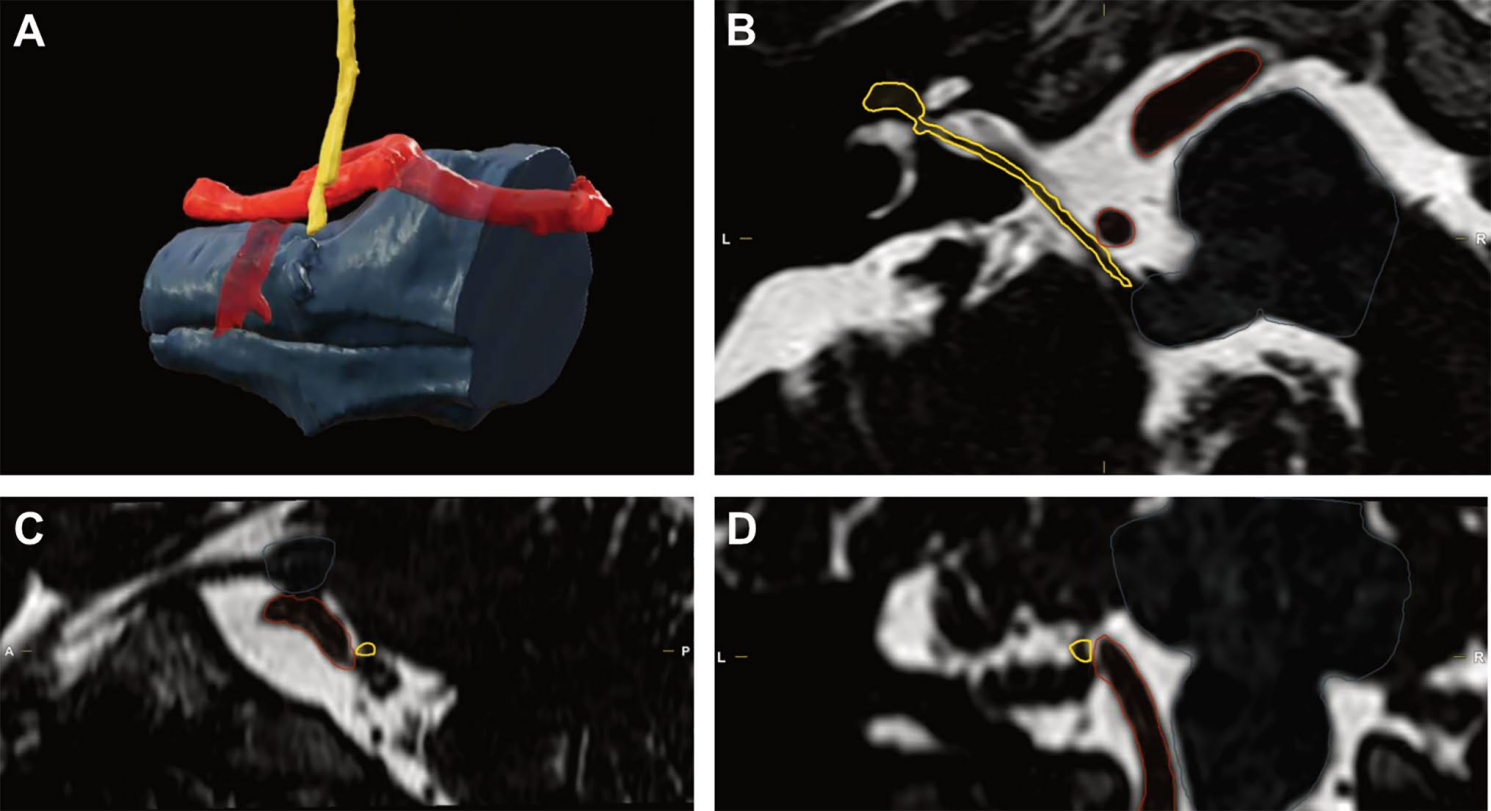
Heavily weighted T2 images for a HFS patient, showing the facial nerve (yellow), relevant blood vessels (red), pons and medulla (gray). The neurovascular conflict is from the ipsilateral vertebral artery. **A** is a 3D reconstruction, created using BrainLab Imaging Software. Images **B**, **C** and **D** display axial, sagittal and coronal sections respectively

As previously mentioned, part of the inadequate response to initial surgery can be related to overreliance on pre-operative MRI reporting. NVCs involving the posterior-inferior cerebellar artery and vertebral artery can be over-described on initial reporting, whereas conflicts involving the anterior-inferior cerebellar artery may be more difficult to visualize and can lead to an underestimation [66]. Figure 1 shows an example of efficient pre-operative planning and assessment of NVC on heavily weighted T2 images.

An endoscopic approach to decompression, which allows systematic exploration of the cerebellopontine angle, in conjunction with a review of the patient’s imaging has also recently been advised by some surgeons to look extensively for possible NVCs [67]. This adds to further adjunctive measures that have previously been suggested to improve outcomes following MVD, such as intra-operative neurophysiology monitoring of lateral spread responses and intra-operative monitoring of brainstem auditory evoked potentials [12, 26, 68–74].

### Miscellaneous treatments

Further treatments that have previously been suggested for HFS, typically in the form of isolated case reports, include pulse radiofrequency ablation (using a maximum temperature of 42 °C for 120 s), which was shown to reduce spasm frequency and severity on a visual analogue scale [75]. However, a recent study showed the durability of this intervention is very short and that it also resulted in high rates of disfiguring facial paresis [76]. Electroacupuncture (2 Hz and ~ 1–2 mA intensity used for two periods over 30 weeks) was also attempted in a recent case report, leading to reduced Jankovic Rating and visual analogue scale scores in an HFS patient, although symptoms gradually reappeared at the 6-month follow-up [77]. These treatments may offer further alternative options to patients who are non-responsive, intolerant or unsuitable for botulinum toxin injections and MVD; however further larger-scale studies are required to assess their true efficacy and safety.

## Conclusion

Recent studies provide further characterization to the NVC that often underpins HFS, as well as possible mechanisms. MRI remains an invaluable tool to search for culprit regions of NVC, although it is important to remember that this relies on the interpretation of the scans by experienced clinicians. Recent studies propose that this could be further aided by 3D MRI techniques, especially 3D-TOF MRA and 3D-FIESTA, to improve the accuracy of reporting and ultimately patient outcomes. The clinical utility of botulinum toxin and MVD remain well validated, however further investigations are warranted to confirm their long-term efficacy. Alternative management options remain mildly efficacious at best, hindered further by unwanted side effects, but still represent potential treatment avenues for patients who are non-responsive, intolerant or unsuitable for botulinum toxin injections and/or MVD.

## Data Availability

Data availability is not applicable to this article as no new data were created or analyzed in this study.
